# Dendritic Cell Regulation by Cannabinoid-Based Drugs

**DOI:** 10.3390/ph3082733

**Published:** 2010-08-23

**Authors:** Mattias Svensson, Puran Chen, Oscar Hammarfjord

**Affiliations:** Center for Infectious Medicine, Department of Medicine, Karolinska Institutet, Karolinska University Hospital, 141 86, Stockholm, Sweden; E-Mails: puran.chen.440@student.ki.se (P.C.); oscar.hammarfjord@ki.se (O.H.)

**Keywords:** Cannabinoids, dendritic cells, inflammation, immunomodulation, K_V_-channels

## Abstract

Cannabinoid pharmacology has made important advances in recent years after the cannabinoid system was discovered. Studies in experimental models and in humans have produced promising results using cannabinoid-based drugs for the treatment of obesity and cancer, as well as neuroinflammatory and chronic inflammatory diseases. Moreover, as we discuss here, additional studies also indicates that these drugs have immunosuppressive and anti-inflammatory properties including modulation of immune cell function. Thus, manipulation of the endocannabinoid system *in vivo* may provide novel therapeutic strategies against inflammatory disorders. At least two types of cannabinoid receptors, cannabinoid 1 and cannabinoid 2 receptors are expressed on immune cells such as dendritic cells (DC). Dendritic cells are recognized for their critical role in initiating and maintaining immune responses. Therefore, DC are potential targets for cannabinoid-mediated modulation. Here, we review the effects of cannabinoids on DC and provide some perspective concerning the therapeutic potential of cannabinoids for the treatment of human diseases involving aberrant inflammatory processes.

## 1. Introduction

Human and animal cannabinoids belong to the group of endocannabinoids that are cell membrane-derived signaling molecules formed during the metabolism of eicosanoid fatty acids [1]. Endocannabinoids, such as anandamide, can activate a group of G-protein-coupled receptors, cannabinoid receptors 1 (CB_1_) and 2 (CB_2_) [2]. The discovery of this 'endogenous cannabinoid system' has led to the development of selective CB_1_ and CB_2_ ligands and fuelled renewed interest in the clinical potential of cannabinoids. Cannabinoids derived from plants (e.g., marijuana) and synthetic structural analogues of cannabinoids referred to as exogenous cannabinoids, can also activate cannabinoid receptors, normally activated by endocannabinoids [3]. In the nervous system, CB_1_ signaling modulates K^+^ and Ca^2+^ channels [4,5,6,7,8]. This CB_1_-mediated modulation of voltage-gated potassium channel (K_V_-channel) functions can be regulated by both endogenous and exogenous cannabinoids [5,8]. Although, the main functions of the endocannabinoid system include neuromodulation, regulation of motor functions, cognition, emotional responses, and motivation, it has become evident that in the periphery, this system is also an important modulator of the immune system. The anti-inflammatory effects are mediated, at least partially, through the binding of cannabinoid receptors and correlate with decreased T cell responses and reduced production of inflammatory mediators [9,10,11]. This results in the attenuation of symptoms and disease progression of neuroinflammatory disorders and inflammatory bowel diseases. Although, a wealth of information indicates that cannabinoids have immune suppressive and anti-inflammatory activities, the exact mechanisms by which cannabinoids exert functional regulation of immune cells, such as DC, need to be investigated in more detail. 

DC are widely distributed immune cells important in innate and adaptive immunity, and in the maintenance of tolerance. By responding to microbial and inflammatory stimuli DC undergo a process of cellular activation termed maturation [12]. Upon maturation, DC transiently increase their capacity to internalize antigen, that is followed by increased migration and enhanced antigen-presenting and T cell stimulatory capacity. This includes changes to their surface phenotype involving redistribution of MHC-II from intracellular compartments to the plasma membrane [13,14,15,16,17]. Maturation is also associated with increased surface levels of co-stimulatory molecules and enhanced production of soluble inflammatory mediators [18,19,20,21]. Consequently, mature DC possess important properties for activating and directing functional differentiation of antigen-specific T cells [22]. Regulation of the immunostimulatory capacity of DC is therefore a key step determining the nature and effectiveness of T-cell-mediated immune responses. In addition, various mechanisms may act at distinct levels in tuning DC function to prevent excessive immune responses and the onset of pathophysiological conditions. Targeting such mechanisms may serve as means for therapeutic modulation of DC function in chronic inflammatory diseases associated with pathological T cell responses, e.g., autoimmunity and transplantation [23]. Studies from our laboratory have suggested that endocannabinoids modulate DC-T cell stimulatory capacity by reducing DC surface expression of major histocompatibility complex class II molecules (MHC-II) [24]. Cannabinoid-treated DC also showed altered K_V_-channel function. Thus, manipulation of the endocannabinoid system *in vivo* may constitute a novel treatment modality against inflammatory disorders. In this article, we have reviewed the possible mechanisms of the regulation of the immune response by endocannabinoids, which include modulation of DC and down regulation of antigen presenting and T cell stimulatory capacity.

## 2. The Endocannabinoid System in Immunity and Inflammation

Endocannabinoids affect diverse biological processes, including functions of the immune system. With respect to the immune system, mainly anti-inflammatory and immunosuppressive effects of endocannabinoids have been reported [11]. The endocannabinoids stimulate G-protein-coupled CB_1_ and CB_2_ [2]. These receptors are found on immune cells and, although the expression levels of CB_2_ in immune cells are 10–100 times greater than CB_1_ both receptors are present on most immune cells, including DC [25,26]. Other cannabinoid receptor types may also exist and the endocannabinoid, anandamide, not only acts through CB_1_ and CB_2_, but is also a vanilloid receptor agonist and some of its metabolites may possess yet other important modes of action [3]. Although, immune cells, such as DC, express both CB_1_ and CB_2_, secrete endocannabinoids and have functional cannabinoid transport and catabolism, the exact role of the CB_1_ and CB_2_ are proving more difficult to establish but seem to include the modulation of cytokine release [27] and immune stimulatory capacities of DC [24,27]. 

Microbial pathogens that invade the tissues are recognized by host cells and host factors that triggers the activation of both innate and adaptive immune responses. Activation of the inflammatory response to infection largely depends on the release of proinflammatory cytokines and chemokines. In addition to cytokines and other proteins, also various host derived metabolic products including membrane fatty acids, such as arachidonic acid have been implicated in the inflammatory response to infection [28]. It is therefore not surprising that chemically similar metabolites such as the endocannabinoid, anandamide, is produced and released in response to inflammation [25,29,30]. In addition, it was reported that cannabionoid receptors on immune cells are activated after infection or immune stimulation. The consequences of this for the immune response are not fully understood, but may involve the regulation of immune cell chemotaxis. However, by the modulation of T and B lymphocytes proliferation and apoptosis, macrophage-mediated killing of sensitized cell, inflammatory cytokine production, immune cell activation by inflammatory stimuli, chemotaxis and inflammatory cell migration, it is evident that endocannabinoids have important effects on the immune system [11]. The immune suppressive effect of endocannabinoids on immune cells has primarily been considered to be mediated through CB_2_ by decreasing the expression of cAMP-responsive genes [26]. The anti-inflammatory effects of endocannabinoids may also be mediated through the activation of peroxisome-proliferative-activated receptor-γ (PPARγ) [31], a member of the nuclear receptor family that regulates the transcription of genes involved in regulating inflammatory processes. 

In both experimental models and human cell cultures it has been demonstrated that cannabinoids suppress the production of cytokines important in innate and adaptive immune responses [11,32,33]. The suppressive role of cannabinoids on proinflammatory cytokine and chemokine production indicates that these drugs might have anti-inflammatory effects and could therefore be used for the treatment of chronic inflammatory diseases. Consistent with this, serum levels of tumour-necrosis factor (TNF) and interleukin-12 (IL-12) were shown to be decreased in mice that were infected with *Corynebacterium parvum* followed by the injection of LPS and then treated with the exogenous cannabinoid, WIN55,212-2 [34]. In this model, cannabinoids also protected mice from a lethal effect of LPS, and this protection might have resulted, at least partially, from a concomitant drug-induced increase in the levels of the regulatory cytokine IL-10. In another mechanistically complex mouse model, treatment with WIN55,212-2 decreased tissue damage after cardial infarct injury. Treatment of animals before ischaemia and reperfusion considerably reduced the size of the infarct, and this was paralleled by lower levels of production of IL-1β and CXC-chemokine ligand 8 (CXCL8) in the injured tissue [35]. Although, most studies on the immunomodulatory effects of cannabinoids point towards immune suppressive properties, cannabinoids have also been shown to increase the production of proinflammatory cytokines when administered together with bacteria [36,37] as well as when cannabinoids are administered alone [38,39]. It therefore seems that cannabinoids have several mechanisms of action and that cannabinoids either suppress or enhance the production of proinflammatory cytokines. However, further investigations aiming at elucidating molecular mechanisms underlying the immunomodulatory effects of CB_1_ and CB_2_ activation are needed.

Recently, it has also been found that anandamide is capable of providing feed-back to control activated microglia and promote neuroprotection in the CNS [40]. Interestingly, peripheral neurons also produce anandamide, and areas of direct communication between neurons and immune cells lie in both primary and secondary tissues of the immune system, as well as airway epithelium and skin [41]. Functionally, neurotransmitters including catecholamines and acetylcholine as well as neuropeptides including CGRP, vasoactive intestinal peptide, somatostatin, SP and pro-opiomelanocortin-derived peptides have been shown to modulate inflammatory responses [42]. There is also evidence indicating that direct innervations may control immune responses [43], and the term “neuro-immunological synapse” has been proposed for contacts between neurons and antigen-presenting cells [44]. Langerhans cells, a subtype of DC that were originally thought to originate in the nervous system because of their close contacts with nerve fibers [45], have recently been linked closely to CGRP/SP-containing fibers in the skin (presumably nociceptive neurons) of humans [46,47,48], primates [46] and rodents [46,49] as well as in the viscera [50]. These same CGRP/SP-containing primary afferent fibers express and release the immune-regulatory endocannabinoids. Specifically, Ahluwalia and coworkers [51] showed that stimulation of capsaicin-sensitive primary sensory neurons induces release of anandamide. Our recent finding that cannabinoids can modulate the activity of voltage-dependent K^+^ currents in DC, therefore, tallies with the hypothesis that neuro-immune interactions may occur at the level of the nerve fiber-immune cell interface. 

## 3. Cannabinoids and Modulation of Dendritic Cell Function

Dendritic cells play an important role in the immune system as regulators of tolerance to self and inducers of immunity to non-self. Therefore, DC are considered ideal therapeutic targets for pharmacological modulation of immune responses [23]. Given that DC express both the CB_1_ and CB_2_, DC may serve as targets for cannabinoid-based drugs. Although, there have been reports indicating that the endocannabinoid system is important in regulating DC biology (Table 1), detailed studies addressing the effects of cannabinoids on cellular processes in DC are relatively scarce. In macrophages, however, it was shown that cannabinoids affect phagocytosis, NO- and cytokine production as well as the capacity to process and present soluble peptide antigens [52,53,54,55,56]. Although, not formely proven, it is possible that cannabinoids affect DC functions in similar ways to that reported for macrophages.

**Table 1:** Effects of cannabinoids on DC.

Source	Functions affected/receptors involved	References
*In vivo*		
Tissue resident DC, murine	2-AG-induced migration from periphery to draining lymph nodes, CB_2_-mediated.	[57]
Spleen DC, murine	THC induced apoptosis, CB_1_- and CB_2_-mediated.	[58]
*Ex vivo*		
Spleen DC, murine	Reduced MHC-II expression and T cell stimulatory capacity, CB_1_-mediated.	[24]
Spleen DC, murine	Increased cell-mediated immunity in response to low levels of anandamide.	[59]
*In vitro*		
Bone marrow-derived DC, murine	Reduced MHC-II expression and T cell stimulatory capacity, CB_1_-mediated.	[24]
Bone marrow-derived DC, murine	2-AG-induced migration, CB_2_-mediated.	[57]
Monocyte derived DC, human	T helper cell type 2-polarized response, CB_2_-mediated.	[60]
Bone marrow-derived DC, murine	THC stimulation induces T helper cell type 1-polarized response and inhibits upregulation of CD86, CD4, and MHC class II.	[27]
Bone marrow-derived DC, murine	DC capacity to kill intracellular Legionella pneumophila is unaffected by THC-stimulation.	[27]
Bone marrow-derived DC, murine	THC induced apoptosis, CB_1_- and CB_2_-mediated.	[58]

In addition, it has been shown that DC produces the cannabinoids AEA and 2-AG at steady state and increase their production when activated with LPS [25]. Thus, DC may not only be affected by cannabinoids themselves but DC may also provide cannabinoid-mediated regulation of other cells. In order to better understand the complexity and the role of DC activities with respect to the endocannabinoid system, additional studies are needed. 

A central part in DC biology is their ability to migrate from peripheral tissues to draining lymph nodes where they elicit and regulate immune responses [61]. Results from *in vitro* studies using murine DC have demonstrated that the endocannabinoid 2-AG induce migration of bone marrow derived DC in transwell-based assays. Furthermore, this study demonstrated that 2-AG, when injected subcutaneously into hind paws of mice, stimulated tissue resident DC to migrate from the periphery to the poplitelial lymph node [57]. In contrast, anti-migratory effects of cannabinoids on cell types other than DC have been presented. For example, recent work by Ramer *et al.* [62] demonstrated that migration of human trabecular meshwork cells is decreased in response to cannabinoids. This may be one possible mechanism by which cannabinoid-based drugs have antiglaucomatous effects. In summary, these data points towards the possibility of using of cannabinoid-based drugs to modulate migration of immune cells, including DC, *in vivo*. 

**Figure 1:**
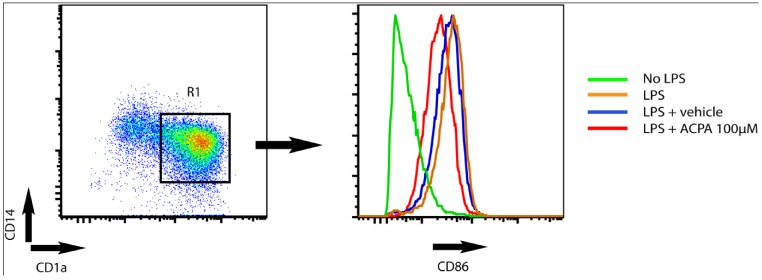
Arachidonylcyclopropylamide (ACPA) reduce LPS-induced upregulation of CD86 surface expression in human monocytes derived DC. The dot plot shows CD1a and CD14 expression on human monocyte derived DC (left). DC were defined as cells being positive for CD1a (CD1a^+^) and negative for CD14^-^ (CD14^-^). The histogram (right) shows CD86 expression on DC (CD1a^+^CD14^-^) within the R1 gate. DC were cultured in medium only (green) or stimulated with LPS for 24 hours in the absence (orange) or presence (red) of ACPA or vehicle (blue, diluent of ACPA. Tocrisolve). ACPA was used at 100 μM.

When the DC reaches the draining lymph nodes, DC orchestrate the immune response by activating and stimulating T cells to proliferate into distinct polarized subpopulations. Studies from Yuan *et al.* [60], showed that the capacity of DC to stimulate T cell proliferation is decreased in the presence of the cannabinoid delta-9-tetrahydrocannabinol (THC). Moreover, these authors showed that THC skews the Th1/Th2 balance in favour of a Th2 polarized response via a CB_2_-dependent pathway. In line with this Lu *et al.* [27] showed that THC inhibits Th1 activation by targeting DC functions, *i.e.*, IL-12p40 secretion, expression of MHC class II, and decreased costimulatory molecule up-regulation. Recent studies from our group demonstrated that endocannabinoids attenuate K_V_-channel function via CB_1_ signalling, leading to reduced surface expression of MHC class II molecules and decreased capacity to stimulate T cells [24]. In addition, we found that the CB_1_ agonist arachidonylcyclopropylamide (ACPA) decrease LPS-induced upregulation of CD86 in human monocyte-derived DC (Figure 1). 

In contrast, it was recently shown that injection of a low dose of the cannabinoid (0.1 mg/mL) anandamide into mice promotes upregulation of CD80 and CD86 costimulatory molecules on DC when stimulated *in vitro* [59]. Furthermore, Do and co-workers [58] showed that treatment of mice with a higher dose (10–50 mg/kg) of exogenous as well as endogenous cannabinoids induces apoptosis in splenic DC. Although, these studies were all performed in murine model-systems, they collectively suggest that the cannabinoid treatment might be used to modulate DC immunostimulatory capacities and subsequently the capacity to stimulate T cell activation, proliferation and polarization. However, more detailed analyses including clinical samples as well as studies on dose response outcomes are needed. 

## 4. Potassium Channel Function as a Target for Cannabinoid Receptor-mediated Modulation of Dendritic Cell Function

Since both the CB_1_ and K_V_-channel are present on lymphocytes, macrophages and DC [63,64,65], the anti-inflammatory effects of cannabinoids might involve regulation of K^+^ channels via CB_1_ signaling in these cells [63,64,65,66,67]. In T lymphocytes, K_V_1.3 channels are inactivated by hypoxia [68] and inhibited by the immunosuppressors cyclosporin, rapamycin and FK-506 [65]. Inhibition of these channels reduces T-cell proliferation and activation and redirects cytolytic activity and cytokine production [69]. Previously it was also demonstrated that blocking of K_V_-channels in human monocyte-derived DC suppresses LPS-induced up-regulation of DC markers of maturation, *i.e.*, the co-stimulatory molecules CD83, CD80, CD86 and the proinflammatory cytokine IL-12 [67]. It was also demonstrated that the K^+^ channels K_V_1.3 and K_V_1.5 are highly expressed on myeloid cells, including DC, in brain tissue of MS patients. In contrast control brain tissue did not show high expression of these K^+^ channels, indicating that K_V_1.3 and K_V_1.5 channels are mainly expressed on infiltrating inflammatory cells or are induced in response to inflammation [67]. In line with this, *in vitro* patch-clamp analysis demonstrated expression of K^+^ channels on human monocyte-derived DC [67], often referred as inflammatory DC. In our laboratory we observed that K^+^ currents are the predominant outward voltage-activated currents in murine bone marrow-derived DC [24]. We also found that the selective activation of CB_1_ in DC, using either exogenous or endogenous cannabinoids, attenuates the voltage-activated K^+^ currents in a time-dependent manner. This attenuation of K_V_ channel function in response to the selective CB_1_ agonist, ACPA, was completely abolished in the presence of a CB_1_ antagonist. Similarly, uncoupling of G-protein signaling by PTX abolished the ACPA-mediated attenuation of K_V_ channel function. Together these findings demonstrated that cannabinoid-regulated K_V_ channel function in DC is mediated via CB_1_ signaling, and not by other noncannabinoid-like receptors (37–40). Importantly, we demonstrated that cannabinoids attenuate K_V_-channel function in DC, reduce the expression of MHC-II surface molecules, and decrease the capacity to induce T cell proliferation. Thus, our findings, together with the recent report that blockade of K_V_-channels can reduce LPS-stimulated up-regulation of maturation markers in human blood-derived DC [67], add to the numbers of targets on which K_V_-channel inhibitors, such as exogenous and endogenous cannabinoids, can exert immunosuppressive effects. 

Our results showed that the K_V_-channel function in DC can be modulated by both exogenous and endogenous cannabinoids through the CB_1_, in a PTX-sensitive manner. Thus, this discovery presents a mechanism by which cannabinoids can regulate DC and may contribute to cannabinoid-induced immunosuppression. Given the important role of K_V_-channels in T cell function, the concept that DC are similarly regulated by K_V_-channel modulation is interesting in the light of DC as targets for immunosuppressive drugs [23]. These results also indicate a potential mechanism by which peripheral neurons may influence the function of DC through the release of the endogenous cannabinoid, anandamide. The down-modulation of MHC-II on DC observed in response to blocking of K_V_-channels may explain some of the immunosuppressive effects mediated by cannabinoids. Since CB_1_ signaling attenuates K_V_-channel function in DC, the CB_1_ can be a potential target to regulate DC function, preventing DC-mediated inflammation and inducing beneficial immunosuppression. Together, these findings support a general model whereby both endogenous and exogenous cannabinoids affect immune cells function and that there seems to be a role for K_V_-channels in regulating immune cell activation. 

## 5. Prospects of Using Cannabinoid-Based Drugs in Immune Therapy

The cannabionoid receptors and their ligands have become an area of great interest in pharmacology due to their capacity to modulate several physiological systems, including modulation of the immune system [11]. Furthermore, studies have suggested that administration of endocannabinoids or use of inhibitors of enzymes that breakdown the endocannabinoids, leads to immunosuppression and organ recovery after immune-mediated injury [70]. In oncology, cannabinoids have been given to patients since the early 1970s due to the antiemetic effects [71]. Other potential therapeutic uses of cannabinoid receptor agonists include the management of diseases, such as multiple sclerosis [72]. Regarding the immune system the administration of cannabinoids mainly causes inhibitory effects on immune cells and this include, decreased cytokine production an proliferation as well as antigen presenting cell-T cell stimulatory capacities [11]. The inhibitory effect on lymphocyte proliferation, NK cell and macrophage activity as well as antibody production and diminished bacterial and viral resistance can partially be explained by the modulation of cytokine production by different immune cells, which in turn serves as a potential targets in different inflammatory disorders as well as in cancer therapy.

*Cannabis sativa*, also known as marijuana, is the classical cannabinoid that have been recognized for centuries to have therapeutic effects, and has been recommended as an analgesic, muscle relaxant and appetite stimulant. During the early twentieth century its therapeutic applications grew to include the use to treat symptoms of a wide spectrum of diseases, ranging from rheumatism to infections, such as gonorrhea. Despite the popularity of marijuana as a therapeutic, its usage declined during the mid twentieth century as the potential for abuse was recognized. However, the view of marijuana and its potential as a therapeutic agent now seems changing with several publications reporting positive effects of marijuana based-drugs for the treatment of disorders including obesity, cancer, neuropathology, and chronic inflammatory bowel disease [71,73,74,75,76] (Table 2). In addition, human lung alveolar macrophages removed from marijuana smokers were compromised in their ability to produce TNF, granulocyte/macrophage colony stimulating factor and IL-6 in response to LPS stimulation [77]. The main psychoactive component of marijuana is the classical cannabinoid THC. 

**Table 2:** Disorders in which cannabinoids have been used as treatment.

Disorder	Cannabinoid	Action	Reference
Lung cancer	THC	Inhibition of tumor growth, metastasis and vascularisation of A549 xenografts with possible involvement of attenuated EGF downstream signalling	[78]
Glioma	WIN 55,212-2, THC	CB receptor-mediated apoptosis by accumulation of ceramide and Raf1/ERK activation	[79]
Breast cancer	Cannabidiol (CBD)	Reduced tumor aggressiveness by decreasing Id-1 expression	[80]
Pancreatic cancer	THC	Iincreased apoptosis by CB_2_-mediated ceramide-dependent upregulation of stress protein p8	[81]
Melanoma	WIN 55,212-2, JWH-133	Inhibition of cell growth, partially owing to cell cycle arrest in G1-S phase by inhibition of Akt	[82]
ALS	Cannabinol (CBN)	Delays disease onset, but no affect on survival in SOD1 mouse model of ALS	[83]
Prostate cancer	WIN-55,212-2	Inhibition of cell growth (cell cycle arrest in G0-G1 phase) and induction of apoptosis by ERK1/2 activation	[84]
Colitis/IBD (chemically induced)	HU-210	Decrease colonic inflammation mediated through CB_1_receptor	[85]
Cancer	HU-331	CB_1_/CB_2_-independent inhibition of topoisomerase II	[86]
Myocardial I/R injury	WIN 55,212-2	CB_2_-mediated protection, parallel with lower levels of IL-1b and CXCL8	[35]
Breast cancer	JWH-133, WIN 55,212-2	CB_1_/CB_2_ mediated inhibition of cell proliferation and migration	[87]
Lymphoma/leukemia	HU-210, THC, JHW-015	Partial CB_2_-mediated apoptosis	[88]

The immunomodulating effects of cannabinoid administration are complex and depend on factors such as dose, disease model and choice of cannabinoid and, although, cannabinoids mainly have immunosuppressive effects, also immunostimulatory effects have been reported. For example THC exerts a biphasic action on proinflammatory cytokine production in peripheral blood mononuclear cells such that TNF, IFNγ, IL-6 and IL-8 production are inhibited in nanomolar concentrations but increased when exposed to micromolar concentrations [89]. Also, decreased levels of anti-inflammatory cytokine IL-10 is seen during exposure to micromolar concentrations of THC, suggesting an anti-inflammatory role for THC in high concentrations. In addition, THC inhibits T cell proliferation and favors release of Th2 (IL-4) and anti-inflammatory (IL-10) cytokines, respectively, that attenuates Th1-mediated immunity to *Legionella pneumonia* [36,60]. Thus, it seems that cannabinoids, at least under some circumstances, bias the immune response away from Th1 immunity and it is possible that signaling through cannabinoid receptors on lymphocytes and antigen presenting cells, such as macrophages and DC suppress production of Th1-promoting cytokines and increase the production of Th2 or immunosuppressive cytokines. The ability to selectively administer and suppress Th1-immunity provides the possibility of using cannabinoids in new strategies for treatment of autoimmune diseases. However, due to the complexity regarding immunomodulatory effects of cannabinoids it is necessary to establish the precise mechanisms of cannabinoid mediated actions on the immune system and its constituent cells. 

Recent applications of cannabinoids include their potential use as anti-tumor drugs and it has been reported that cannabinoid induced signaling is differentially regulated in cancer cells *versus* normal cells (Table 2). In malignancies, such as lymphoma, melanoma and breast cancer, the levels of cannabinoid receptors are often higher in the tumors as compared to normal cells, resulting in increased sensitivity to cannabinoids. Moreover, cannabinoids have anti proliferative and pro-apoptotic effects on tumor cells but not on normal tissue [79,81,87]. In contrast, the cannabinoid, THC, has been shown to enhance breast cancer growth and metastasize in mice injected with 4T1 tumor cells by suppressing specific antitumor immune response *in vivo* [90] as well as suppress host immune reactivity in a CB_2_-dependent manner against lung cancer cells *in vivo* [37]. This was, in part, explained by elevated levels of IL-4 and IL-10 as well as TGFβ since inhibition of these cytokines reversed the immunosuppressive effect of THC. Taken together, these data suggests that THC exposure could increase the susceptibility to and/or incidence of breast cancer as well as other cancers controlled by Th1- rather than Th2-mediated immunity. While Th1 responses are considered antitumorogenic and Th2 responses favors tumor growth by inhibiting Th1 cell-mediated immunity as well as promoting angiogenesis, there are also studies demonstrating anti-tumor activity of Th2 responses. Antigen specific CD4^+^ Th2 cells, and not Th1, have been shown to be able to eradicate B16 melanoma-induced metastases [91]. This Th2 cell dependent clearance was attributed to the eosinophil chemokine, eotaxin, STAT6 and degranulating eosinophils in the tumor. The hallmark Th2 cytokine IL-4 has also been reported to inhibit corneal neovascularisation in rats [92], however, a recent report showed that chemokines, which are considered pro-angiogenic, are induced in human microvascular endothelial cells during Th2 inflammation rather than Th1 inflammatory conditions *in vitro* [93]. Interestingly, chemokines and their receptors have recently been shown to act at all stages of tumor development, including neoplastic transformation and the promotion of aberrant angiogenesis. Examples include CXCL1, CXCL2, CXCL3, CXCL5 and CXCL8. Chemokines are produced by a variety of cells, including DC that contribute to the coordinated expression of chemokines in tissue and either produce cytokines themselves or induce specific tissue cells, such as epithelial cells, to produce chemokines. The precise cellular components and niche derived factors regulating chemokine production in healthy tissue and in the course of tumor progression remains unclear. However, a growing body of literature suggests that such instructive processes are mediated by the interplay between tissue specific cells and immune cells. For example, stimulation of lung epithelial cells with IL-1β derived from monocytes, macrophages and DC could contribute to chemokine production of angiogenic factors, such as vascular endothelium growth factor (VEGF) and chemokines including CXCL5 and CXCL8 [94]. Nevertheless, given the importance of chemokines in tumor progression, interfering with chemokine production capacities in tissue by cannabinoid-mediated modulation of DC function can provide new opportunities for cancer prevention and treatment.

Interesting hypotheses have been made regarding the dose-dependent mechanism of immune modulation. At low cell density (1 × 10^6^ cells/mL), 2-AG inhibited anti-CD3 and LPS induced lymphoproliferation, whereas at high cell density (>5 × 10^6^ cells/mL) 2-AG had reversed effect [95]. This effect was not due to “dilution” of 2-AG, instead, the authors hypothesize that 2-AG is rapidly metabolized in high density cell cultures to immunestimulatory by-products. Cannabinoids that mediates immunosuppression could therefore be the result of saturation of degradation enzymes, a possible target for modulation of the endocannabinoid system and its functions. Efforts to restore the balance of the cannabinoid system come with many challenges, especially regarding the selectivity of the modulation favoring improved disease control as well as the diversity of cannabinoid-mediated effects. Antiproliferative effects in breast cancer can be obtained *in vitro* by reduction of FAAH expression, leading to accumulation of its substrate, anandamide [96], but could potentially lead to decreased formation of arachidonic acid, which serves as precursor to many biological active compounds with immunomodulating potential. Furthermore, when increasing the cannabinoid levels, for instance via FAAH-inhibition, other metabolizing pathways should also be considered. For example, anandamide can be a substrate for COX-2 and produce prostamide E2 which have been shown to play a role in inhibiting the activity of IL-12p40 promotor suggesting a mechanism by which anandamide could regulate IL-12p40 gene expression. Understanding the physiological regulation of the endocannabinoid levels and its metabolites in immune modulation have the potential to unveil novel therapeutic targets against inflammatory diseases as well as cancer.

## 6. Concluding Remarks

It is evident that cannabinoids can contribute to DC regulation by the modulation of ion-channel function, in particular that of voltage-gated potassium Kv-channels [24]. The few examples to date of cannabinoid-induced regulation of DC function resulting in the generation of DC with reduced immunostimulatory capacity highlight a previously unrecognised immunoregulatory role for cannabionoids and potassium channels. This may be a mechanism involved in down regulating immune responses to avoid tissue pathologies. Furthermore, the data discussed here suggest that a more complete understanding of the effects of cannabinoids on DC functions should be important new parameters to study when investigating the role of cannabinoids as modulators of inflammation. 

The suppression, by marijuana-based drugs, of the chronic inflammatory response and the subsequent attenuation of disease processes and symptoms are well accepted. These anti-inflammatory effects are undoubtedly, in part, associated with the ability of these drugs to suppress the expression of cytokines, as well as other endogenous proinflammatory mediators. In addition, these drugs might also function by increasing the production of anti-inflammatory mediators. Further analysis of the effect of marijuana-based drugs on proinflammatory and anti-inflammatory mechanisms in human immune cells will provide the basis for the formulation of more effective drugs for the management of chronic inflammatory diseases.

The importance of the cannabinoid system regulation of DC biology, extends beyond chronic inflammation; cannabinoid-mediated regulation of DC might have important physiological and pathophysiological significance in many processes, including vaccinations and immunotherapy, pathogenesis of autoimmunity, angiogenesis, and the progression of tumors. Research focusing on cannabinoid-regulated potassium channel function and the consequences for programming distinct DC functions and possibly differentiation could help to develop new strategies for immune intervention and understanding imbalances of the immune system associated with many human diseases. Therefore, it will be important to identify the specific molecular mechanism underlying the immune-suppressive effects of CB_1_ and CB_2_ activation by both endo- and exocannabinoids in DC. 

A future challenge is to increase our knowledge of where cannabinoids interact with DC within the local tissue microenvironment, particularly in humans. This knowledge can be used to modulate DC functions for therapeutic application in disorders characterised by the loss of tissue homeostasis (*i.e.*, autoimmunity and transplantation). In cancer, the aim might be to block inflammatory pathways that controls and mediate the production of pro-angiogenic factors. However, a more comprehensive understanding of how to administer cannabinoid-based drugs in order to predict the dose that will be both effective and tolerable to a patient as well as the development for better cannabinoid formulations and modes of administration are needed. For the therapeutic potential of cannabis or CB_1_ agonists to be fully exploited, it will be important to establish (*i)* whether the effects of these agents are clinically significant and, if so, whether the benefits outweigh the risks, (*ii*) whether cannabis has therapeutic effects advantageous to individual cannabinoids, (*iii*) whether drugs with reduced psychotropic activity and retained ability to act through CB_1_ can be developed, and (iv) whether it will be possible to develop new strategies aiming at targeting the production and/or metabolism of endocannabinoids in tissue locally. 

## Abbreviations

ACPA: arachidonylcyclopropylamide;
AEA: arachidonylethanolamide;
7AAD: 7-aminoactinomycin;
CB_1_: cannabinoid receptor 1;
CB_2_: cannabinoid receptor 2;
COX-2: cyclooxygenase-2;
CXCL: CXC-chemokine ligand;
CGRP: calcitonin gene-related peptide;
DC: dendritic cells;
FAAH: fatty acid amide hydrolase;
IL-12: interleukine-12;
K_V_-channel: voltage-gated potassium channel;
LPS: lipopolysaccharide;
MHC-II: MHC class II molecules;
NGS: normal goat serum;
PPARγ: peroxisome-proliferative-activated receptor-γ ;
PTX: pertussis toxin;
RT: room temperature;
SP: substance P;
TEA: tetraethyl-ammonium;
TNF: tumour-necrosis factor;
THC: delta-9-tetrahydrocannabinol;
VEGF: vascular endothelium growth factor.
